# An unusual cause of abdominal pain

**Published:** 2015

**Authors:** Shabnam Shahrokh, Mohammad Reza Zali

**Affiliations:** *Gastroenterology and Liver Diseases Research Center, Shahid Beheshti University of Medical Sciences, Tehran, Iran *

## Question

A 26 year old woman was seen in outpatient clinic because of recurrent colicky abdominal pain, epigastric fullness, vomiting and progressive weight loss since 2 months ago, which was exacerbating by eating food and relieved after vomiting. She was admitted several times and got treatment for partial intestinal obstruction which was relieved with conservative treatment only or symptomatic treatment of vomiting and abdominal pain but her disease progressed to more severe pain and vomiting, Progressive weight loss and fewer attacks interval that completely disrupted her life.

**Figure:**
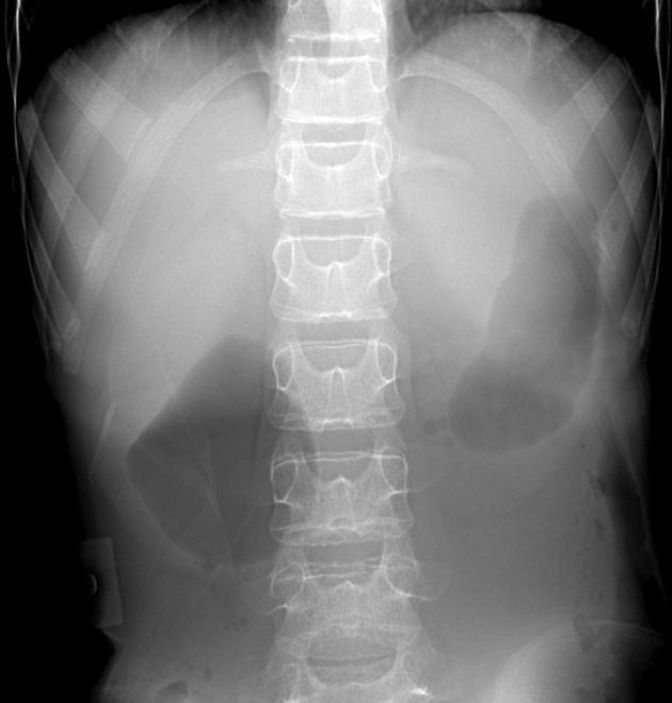
**(A)**

On examination her vital signs were stable, the body mass index was 17 kg/m2, cardiopulmonary examination was normal. On abdominal examination Bowel sounds were present; but the abdomen was mild distended and diffusely tender, but with no rebound or guarding. Pelvic examination results were normal. All laboratory test results were within normal limits. Her previous imaging study was reevaluated.

**Figure:**
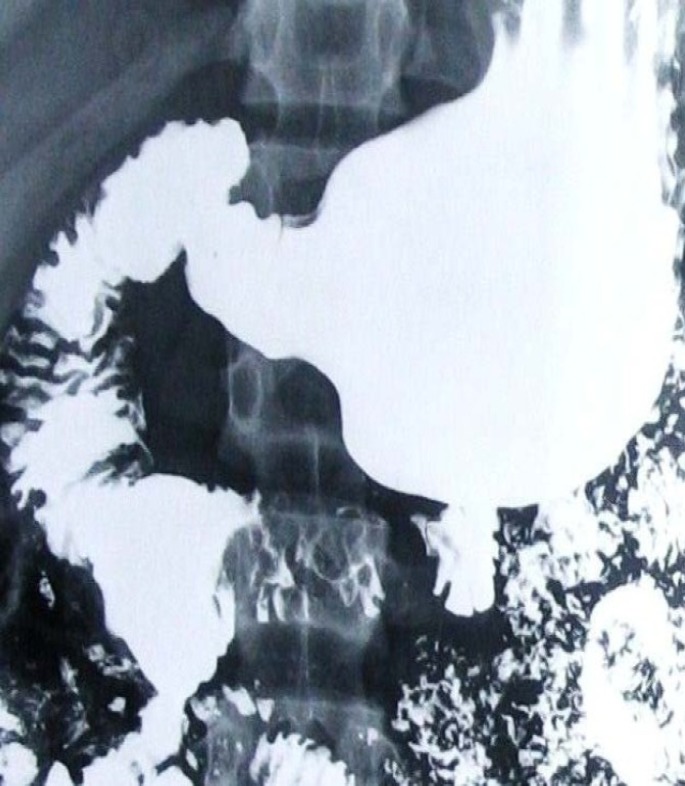
**(B)**

**Figure:**
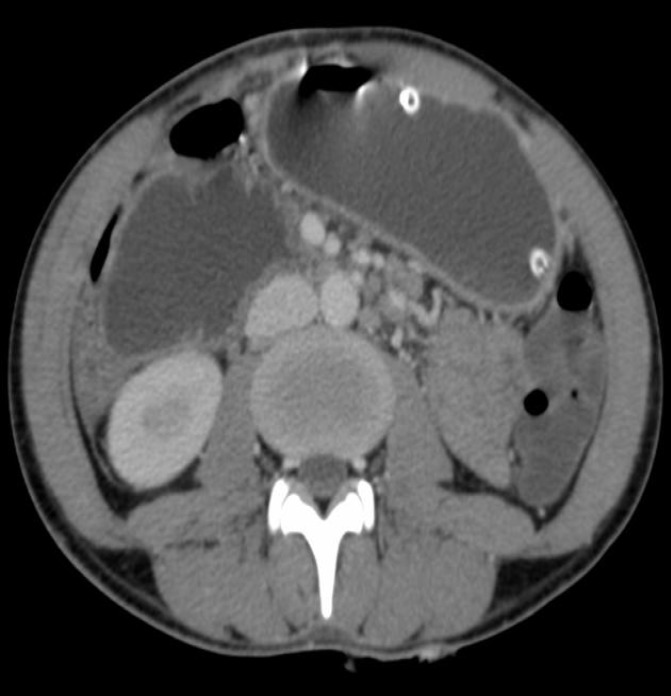
**(C)**

**Figure:**
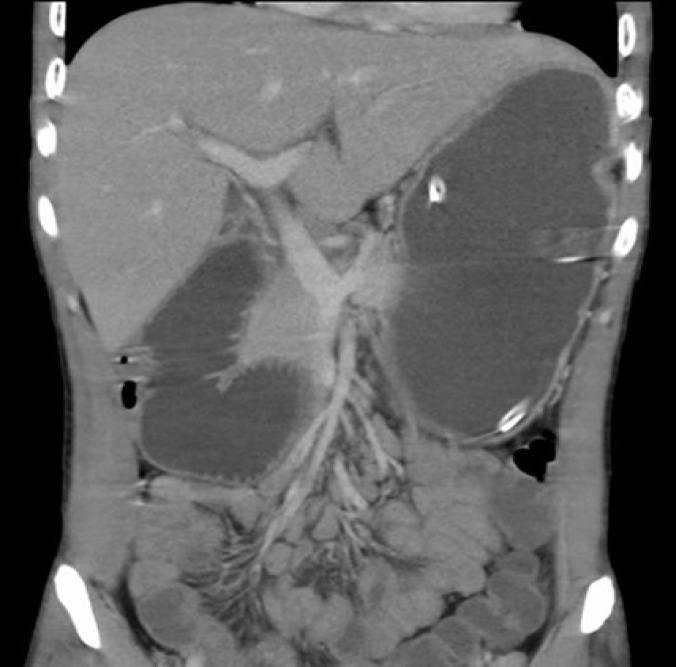
**(D)**

**What is the diagnosis?**

A plain radiograph demonstrates a dilated, fluid- and gas-filled stomach and dilated of proximal of duodenum, probable double bubble sign indicating proximal bowel obstruction (figure A), and barium radiography shows dilatation of the first and second part of the duodenum, extrinsic compression of the third part, and a collapsed small bowel distal to the crossing of the SMA (figure B). 

A computed tomography scan of the abdomen demonstrated dilation of the stomach and proximal duodenum with a transition point at the third portion of the duodenum with no obvious intralumenal, mural, or extrinsic mass (Figure C).

For confirming the diagnosis CT angiography was performed (figure D). The superior mesenteric artery was confirmed on CT angiography to be compressing the duodenum.

## Discussion

Superior mesenteric artery (SMA) syndrome was first described in 1861 by Carl Freiherr von Rokitansky in victims at autopsy, but remained pathologically undefined until 1927 when Wilkie published the first comprehensive series of 75 patients (1). SMA syndrome is an uncommon (incidence of 0.1-0.3%) but well recognized clinical entity characterized by compression of the third, or transverse, portion of the duodenum between the aorta and the superior mesenteric artery (2). The third portion of the duodenum passes normally between the aorta and the superior mesenteric artery that arises from the anterior aspect of the aorta at the level of the L1 vertebral body and enveloped in fatty and lymphatic tissue that provide protection to the duodenum against compression (3). In the majority of patients, the normal angle between the superior mesenteric artery and the aorta is between 38 and 65º and significantly correlate with BMI in normal population (4).

In superior mesenteric artery syndrome, the angle can be narrowed to as low as 6º with the aorto-mesenteric distances as low as 2 mm which minimizes the space between the superior mesenteric artery and aorta potentially leading to duodenal compression. This results in chronic, intermittent, or acute complete or partial duodenal obstruction (5).

The most common factors that decrease the acuity of the angle between the aorta and superior mesenteric artery is significant weight loss leading to loss of the mesenteric fat pad as a consequence of severe, debilitating illnesses, such as malignancy, malabsorption syndromes, AIDS, trauma and burns. It has also been described in a variety of other disorders associated with extreme weight loss including bariatric surgery, spinal cord injury, paraplegia , drug abuse, prolonged bed rest and anorexia nervosa and prolong and severe restriction of diet (6-18). Congenital or acquired anatomic abnormalities can also contribute like following corrective spinal surgery for scoliosis and Rarely, a patient may have a congenitally short ligament of Treitz suspending the duodenum in an abnormally cephalad position or abnormally low origin of the superior mesenteric artery (19-21).

For diagnosis high index of suspicion is required in the appropriate clinical setting. The diagnosis is established in patients with clinical features that suggest duodenal obstruction and noninvasive imaging that demonstrates an abnormal angle between the aorta and the superior mesenteric artery. Diagnostic evaluation should begin with abdominal radiographs which is nonspecific but may reveal findings suggestive of proximal small bowel obstruction such as gastric distension, dilation of the proximal duodenum, and, occasionally, an abrupt vertical cutoff of air in the third portion of the duodenum.

Upper gastrointestinal series usually demonstrate marked delay in passage of the contrast from the duodenum into the more distal small bowel and typically halts abruptly at the third portion of the duodenum (22). Similar findings can be seen with computed tomography (CT). As a general rule, the following criteria should be present on imaging (22-24):

- Duodenal obstruction with an abrupt cutoff in the third portion and active peristalsis.
- An aorto-mesenteric artery angle of ≤25° is the most sensitive measure of diagnosis, particularly if the aorto-mesenteric distance is ≤8 mm.
- High fixation of the duodenum by the ligament of Treitz, abnormally low origin of the superior mesenteric artery or anomalies of the superior mesenteric artery.

Treatment of SMA syndrome included conservative or surgical approach. Medical therapy should be attempted prior to surgery .The goals of conservative treatment of superior mesenteric artery syndrome are alleviation of obstructive symptoms and reversal of any precipitating factors such as gastric decompression, fluid resuscitation and correction of electrolyte abnormalities and nutritional support(Small meal in special positions such as knee chest position, Enteral feedings past the ligament of Treitz or in severe condition parenteral feeding) (25,26) if the patient has altered anatomy the likelihood that conservative therapy will be successful is low, because of that for patients diagnosed with superior mesenteric artery syndrome whose condition is not improved with conservative management, surgical options is considered.

There are several surgical options for the treatment of superior mesenteric artery syndrome that include Strong’s procedure (mobilizes the duodenum by dividing the ligament of Treitz), gastrojejunostomy, and duodenojejunostomy with or without division or resection of the fourth part of the duodenum (25, 26). Laparoscopic treatment with a duodenojejunostomy has been successfully described and may become the standard of care for treatment of SMA syndrome (27).

Our patient was undergo laparoscopic surgery, the diagnosis was confirmed during surgery because of evidence of acute angle between the aorta and SMA artery which possibly was congenital and was exacerbated with strict diet. 

Duodenojejunostomy was performed and she fully recovered after surgery and back to normal life.
